# Supplementary material for: The Evolution and Characterization of the RNA Interference Pathways in Lophotrochozoa
Source: Genome Biol Evol. 2024 May 7;16(5):evae098. doi: 10.1093/gbe/evae098 (PMC11114477; doi:10.1093/gbe/evae098)
Supplement: evae098_Supplementary_Data [file evae098_supplementary_data.zip › Supplementary_materials.zip!/selected_miRNAs/NoGe_selected_miRNAs/novel_miRNA_families_pdf_files/NMRB01001254.1_Notospermus_geniculatus_strain_Ushimado_scaffold1254,_whole_genome_shotgun_sequence_26724.pdf]

Provisional ID	:	NMRB01001254.1_Notospermus_geniculatus_strain_Ushimado_scaffold1254,_whole_genome_shotgun_sequence_26724
Score total	:	113444.2
Score for star read(s)	:	3.9
Score for read counts	:	113435.7
Score for mfe	:	1.6
Score for randfold	:	0
Score for cons. seed	:	3
Total read count	:	222511
Mature read count	:	222441
Loop read count	:	0
Star read count	:	70

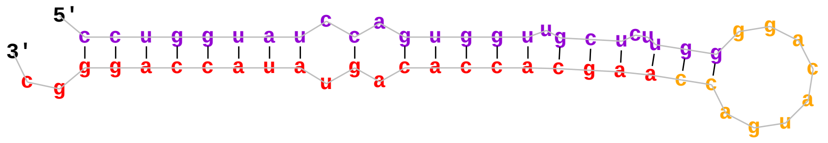

Genome coordinates: NMRB01001254.1 23655-23712

pre-miRNA sequence:
CCTGGTATCCAGTGGTTGCTCTTGGCCTGGTATCCAGTGGTTGCTCTTGGGGACATGACCAAGCACCACAGTATACCAGGGC

Mature sequence: AAGCACCACAGTATACCAGGGC

BLAST results for the pre-miRNA sequence against the *Lineus longissimus* genome assembly:

Scaffold	Start	Stop	Identity	e-value
OU342992.1 1	19328283	19328341	91.525	7.41e-15

```
Nge_novel_miRNA        CCTGGTATCCAGTGGTTGCTCTT--GGGGACATGACCAAGCACCACAGTATACCAGGGC
Llo_genome             CCTGGTATTCAGTGGTTGCTCTTGGGGGGAAATGGCCAAGCACCACAGTATACCAGGGC
```
